# 

**Published:** 1860-10-01

**Authors:** 


### INDEX TO VOL. XIII.

AGAPEMONE, xlviii
Alcoholic Melancholia, Dr. Thomeuf on, 133
Alcoholism, 125.
AMERICA, Young, xxxiii
——— statistics of insanity in, 554
American ships, outrages in, xxxvi
Anthropometamorphosis, Bulwer's, or Artificial Changling, 308
Arlidge, on the asylums of France, &c. Grenoble and Stephansfeld, 335
Auzouy, Dr., on lesions of the cutaneous sensibility among the insane, 68
Asylums, private, Dr. E. Jarvis on, 596
——— patronal, by Drs. Mundy and Bulckens, 602

BAILLARGER, Dr., on doctrines of first writers on general paralysis, 261
Bain, Alexander, A.M. on the senses and intellect, 205
——— on the emotions and will, 205
Belgium, on the management of the insane in, by Dr. J. Parigot, 600
Billod, Dr., on a form of cachexy peculiar to the insane, 425
——— on pellagra in the lunatic asylums of Italy, 606
Bleaching works, state of young persons in, xxxv
Boismond, Dr. Brierre de, on the unity of the human species and amelioration of races, 458
Bouchut, Dr., on nervosism, 218
Braïdism, Dr. Philips on, 516
Brain, consecutive ramollissement of, 272
Broca, Dr. P., on hybridity in man and plurality of human species, 588
Bulckens, Dr., on patronal asylums, 602
Bulwer, John, 294

Cachexy, a form peculiar to the insane, Dr. Billod on, 425
Castelnau, Dr. M. H., on interdiction, 275
Census of 1861, and lunacy, 404
Cerebral ramollissement, consecutive, by Dr. A. Gubler, 273
Chipley, Dr. W. J., on sitomania, 266
Chirologia, Bulwer's, 296
Chironomia, Bulwer's, 300
Chorea, the mental state in, by Dr. Marcé, 271
Civilization, Sir J. K. Shuttleworth on, iii
——— the *Times* on, vi
Committee, select, on lunatics, report of, 408
Cretinism, prize essay on, 613
Crime, status of, in 1859, 484
Criminals, reformation of, the *Times* on, viii
Criminal statistics, French, xxvi
——— English, 484
Criminal Lunatics Act, 586
Criminal responsibility of hysterical individuals, 270
Cullen, Dr., a psychological study, 82

Delirium, a form of hypochondriacal, by Dr. J. V. Marcé, 264
Desmaisons, Dr., on the asylums of Spain, 162
Divorce Court, the moral of, xix
——— the *Times* on, xxiv
Drama, the modern, 502
Dunglison, Dr. on statistics of insanity in America, 554

Epilepsy, Drs. Schröder van der Kolk, Kussmaul, and Tenner on, 241

Fichte the younger, the mental philosophy of, 175
France, the asylums of, by Dr. Arlidge, 335

Gheel, Dr. Parigot on, 600
——— a patronal asylum, Drs. Mundy and Bulckens on, 602
——— Dr. W. Jessen on, 612
Gladstone, Mr., on human progress, xlii

Gladstone, Mr., on student life and duties, xliv
—————— ethical influence of Wine Bill, lii
Grenoble, the asylum of, by Dr. Arlidge, 336
Gubler, Dr. A., on cônsecutive cerebral ramollissement, 272
Gratiolet, Dr. P., on Microcephalus, and the characteristics of the human race, 607
Gundry, Dr., on puerperal insanity, 414

Homicidal maniacs at large, xii
Human progress, Mr. Gladstone on, xliii
Human species, on the unity of, by Brierre de Boismont, 458
—————— characteristics of race of, by P. Gratiolet, 607
Hysteria in connexion with religious revivals, 24
Hybridity in man, Dr. P. Broca on, 588

Insanity, the curability and cost of, by Dr. Reed, 603
—————— lesions of cutaneous sensibility among the insane, 68
—————— music in the treatment of, 100
—————— on a peculiar form of cachexy in, 425
—————— plea of, xviii
—————— puerperal, Dr. Gundry on, 414
Interdiction, on, by Dr. M. N. de Castelnau, 275
Intoxication, on habits of, as causing a type of disease, 125
—————— Dr. Marcet on chronic alcoholic, 130
Ireland, state of lunacy in, 1857-58, 1858-59, 103
Irish exodus, the psychological aspects of, xlvi
Irving, Dr., on a form of paralysis from food, in Allahabad, 407

Jarvis, Dr. Edward, on private asylums, 596
Jessen, Dr. W., on Gheel, 612

Kussmaul, Dr., on epilepsy, 241

Laycock, Dr., on mind and brain, 354
Lévi, Eliphas, Histoire de la Magie, 246
Lewes, Mr. George Henry, physiology of common life: the nervous system, 394
Literature, licentious, xxii
London, moral therapeutics of, lvi
Lunacy, state of, in Ireland, 1857-58, 1858-59, 103
—————— state of, in Scotland, 1858, 377
—————— state of, in England, 1859, 525
—————— the census of 1861, &c., 404
Lunatics, criminal, legislation on, session 1859-60, 586
—————— parliamentary inquiry on, 45, 438
—————— pauper statistics and state of, 109
—————— Mr. Doyle and the commissioners in lunacy on, 109
—————— popular notions on the treatment of, 45
—————— report of select committee on, 438
Lunatic asylums, on the reform of, by Dr. Parigot, 278

Magicians, modern and mediomaniacs, 246
Maniacs, homicidal at large, xvi
Marcé, Dr. L. V., on a form of hypochondriacal delirium, 264
—————— Dr., on the mental state in chorea, 271
Marvellous, the, the modern developments of, 534
Mediomaniacs, 255
Medico-legal trials. *See* Trials.
Melancholia, alcoholic, 123
Mercantile marine, barbarity in, xxxvi
Microcephalus, and the characteristics of human race, Dr. Gratiolet on, 607
Modern Drama, the, 502
Morel, Dr. B.A., on mental disorders, 233
Morel, J. D., on the mental philosophy of Fichte the Younger, 175
Moore, James, case of, xii
Mundy, Dr., on patronal asylums, 602
Music, instrumental, in the asylum of Quatre-Mares, 100

Nervousness, 218
Nervosism, Dr. Bouchut on, 218
Nether-class life, liv

Outrages on board American ships, xxxvi

Paralysis, general doctrines of first writers on, by Dr. Baillarger, 261
—————— of lower extremities from food in Allahabad, 407
Parigot, Dr. J., on reform of lunatic asylums, 278

Parigot, Dr. J., on management of insane in Belgium, 600
Parliamentary inquiry on lunatics, 45, 438
Pathomyotomia, Bulwer's, 302
Pellagra, in the lunatic asylums of Italy, Dr. Billod on, 606
Philocophus, Bulwer's, 300
PINEL, a biographical study, by Dr. Casimir Pinel, 184
Platonic Dialogues, the, Whewell, 144
Popular notions on the treatment of lunatics, 45
Popular physiology, the nervous system, 394
Pownall, Dr., case of, xii
Prayer-meetings, midnight, for street-walkers, xxx
Psychologie Morbide, Dr. J. Moreau on, 12
Psychology, Bain's, 205
——— parodoxical, 1
Psychologist, a medical, of the 17th century, 294
Puerperal insanity, Dr. Gundry on, 414

QUAKERISM, the decadence of, x
Quatre-Mares asylum, music in, 100

REDGRAVE, Mr. Samuel's, judicial statistics, 484
Revivals, religious, hysteria in connexion with, 12

Saulle, Dr. Legrand du, on the criminal responsibility of hysterical individuals, 270
Schröder van der Kolk on epilepsy, 241
Schröder van der Kolk, on the independence of the soul, 314
SCOTLAND, state of lunacy in, 1858, 377
Sheppard, Dr. Edgar, on Quakerism, x
Shuttleworth, Sir J. K., on civilization, iii
Sitomania, Dr. Chipley on, 266
Skipper and Skipper *v.* Bodkin and others, 118
Société Médico-Psychologique, prize essay for 1862 on cretinism, 613
Social evil, the xxix
SPAIN, the asylums of, 162
STATISTICS, French criminal, xxvi
——— of pauper lunatics, 109
——— judicial, 1859, 484
——— of insanity in America, 554
Stephansfeld, the asylum of, by Dr. Arlidge, 339
Student life and duties, Mr. Gladstone on, xliv
Suicide, common-place, lvii

TIMES, the, on civilization, vi
——— on reformation of criminals, viii
TRIALS, MEDICO-LEGAL:—
  Skipper and Skipper *v.* Bodkin and others (disputed will), 118
  Williams *v.* Evans, 433

Volunteer movement, educational tendencies of, xlii

Whewell, Dr., the Platonic Dialogues, 144
Williams *v.* Evans, 433
Wine Bill, the, ethical influence of, lii.

END OF VOL. XIII.

